# Comprehensive characterization of the patient-derived xenograft and the paralleled primary hepatocellular carcinoma cell line

**DOI:** 10.1186/s12935-016-0322-5

**Published:** 2016-06-08

**Authors:** Phyllis F. Y. Cheung, Chi Wai Yip, Linda W. C. Ng, Kwok Wai Lo, Chit Chow, Kui Fat Chan, Tan To Cheung, Siu Tim Cheung

**Affiliations:** Department of Surgery, The Chinese University of Hong Kong, Prince of Wales Hospital, Shatin, Hong Kong, China; Department of Anatomical and Cellular Pathology, The Chinese University of Hong Kong, Hong Kong, China; Department of Surgery, The University of Hong Kong, Hong Kong, China; Division of Genomic Technologies, RIKEN Center for Life Science Technologies, Yokohama, Japan; Department of Pathology, Tuen Mun Hospital, Hong Kong, China; Li Ka Shing Institute of Health Sciences, The Chinese University of Hong Kong, Hong Kong, China

**Keywords:** Hepatocellular carcinoma, Cell line establishment, Patient-derived xenograft

## Abstract

**Background:**

Hepatocellular carcinoma (HCC) is an aggressive cancer with high mortality and morbidity worldwide. The limited clinically relevant model has impeded the development of effective HCC treatment strategy. Patient-derived xenograft (PDX) models retain most of the characteristics of original tumors and were shown to be highly predictive for clinical outcomes. Notably, primary cell line models allow in-depth molecular characterization and high-throughput analysis. Combined usage of the two models would provide an excellent tool for systematic study of therapeutic strategies. Here, we comprehensively characterized the novel PDX and the paralleled primary HCC cell line model.

**Methods:**

Tumor tissues were collected from HCC surgical specimens. HCC cells were sorted for in vivo PDX and in vitro cell line establishment by the expression of hepatic cancer stem cell marker to enhance cell viability and the rate of success on subsequent culture. The PDX and its matching primary cell line were authenticated and characterized in vitro and in vivo.

**Results:**

Among the successful cases for generating PDXs and primary cells, HCC40 is capable for both PDX and primary cell line establishment, which were then further characterized. The novel HCC40-PDX and HCC40-CL exhibited consistent phenotypic characteristics as the original tumor in terms of HBV protein and AFP expressions. In common with HCC40-PDX, HCC40-CL was tumorigenic in immunocompromised mice. The migration ability in vitro and metastatic properties in vivo echoed the clinical feature of venous infiltration. Genetic profiling by short tandem repeat analysis and p53 mutation pattern consolidated that both the HCC40-PDX and HCC40-CL models were derived from the HCC40 clinical specimen.

**Conclusions:**

The paralleled establishment of PDX and primary cell line would serve as useful models in comprehensive studies for HCC pathogenesis and therapeutics development for personalized treatment.

**Electronic supplementary material:**

The online version of this article (doi:10.1186/s12935-016-0322-5) contains supplementary material, which is available to authorized users.

## Background

Hepatocellular carcinoma (HCC) is an aggressive solid tumor with high mortality and morbidity rate worldwide [1]. HCC is frequently diagnosed at advanced stage and therefore curative treatment is not feasible. Chemotherapy only showed marginal efficacy due to the highly chemoresistant nature of HCC [2]. Sorafenib, the only targeted therapeutic agent for HCC, demonstrated only modest improvement in overall survival. Therefore, there is an urgent need to elucidate the pathogenesis and develop new therapeutic strategies for HCC. However, the lack of clinically relevant models has impeded the development of effective HCC treatment strategy [3].The major limitation of conventional cell line models is their poor predictive power on clinical outcome [3]. This is due to the changes in the biological properties of cancer cells during their adaptation to the in vitro culture conditions and long-term culture [4, 5]. Recently, there has been increasing interest in the development of PDX models for improving the drug development process [6–9]. Numerous studies showed that the response rates in PDX models correlated with those observed in the clinic, both for targeted therapeutic agents and for conventional cytotoxic drugs [10–16]. More importantly, remarkable concordance was demonstrated when comparing the responses of individual patients with their corresponding PDX models [15, 17–19]. However, PDX establishment required relatively long period of time (usually 4–8 month) [20, 21], when compared to primary cell line establishment. Therefore, cell line models matching to corresponding PDX models would be needed for high throughput analysis and functional studies. While PDXs reflect the histological and phenotypic characteristics of the original tumors, their matching cell lines could be genetically manipulated to allow in-depth molecular and functional studies and high-throughput drug screening.

Maintaining high viability of the freshly isolated tumor cells is a critical parameter for successful establishment of PDXs and primary cell lines. Pre-operative procedures and hepatocyte isolation often induce extensive necrosis and apoptosis and greatly reduce the cell viability. Therefore, efforts have been made on protocol optimization to increase the cell viability and success rate for cancer models. Isolating and enriching cancer stem cells (CSC) prior to implantation into mice was shown to improve engraftment rates [22]. Previously, we showed that granulin-epithelin precursor (GEP) was a CSC marker in HCC [23], and GEP-expressing cells were resistant to anoikis-induced apoptosis [24]. GEP level in tumor specimen was positively correlated with the viability of freshly isolated hepatocytes and the success rate of subsequent primary culture [24]. In addition, tumor-derived spheroids were found to survive better under in vitro conditions, and could generate tumors when implanted into mice. Besides, these spheroids are composed of pure epithelial cells without non-epithelial lineage cells [25], so that fibroblast contamination and outgrowth can be minimized. Therefore, we attempted to optimize the protocol by enriching GEP-expressing cells for PDX and cell line establishment. For in vitro cell line establishment, we employed our previously optimized protocol [24], as well as the tumor-derived spheroid approach to increase the success rate.

In present study, we described the establishment of a new PDX and matching primary cell line from fresh tumor specimen of HCC patient. A novel PDX model, HCC40-PDX, and its matching primary cell line, HCC40-CL, were established from a patient with early-staged and moderately differentiated HCC. Both models were authenticated by short tandem repeat (STR) analysis and they resembled the genetic and biological characteristics of the original tumor. These established cancer models in early passages would serve as useful tool for studying the molecular pathogenesis of HCC and provide a preclinical tool for therapeutic trial and design.

## Methods

### Specimen collection

The study protocol was approved by the Institutional Review Board of the University of Hong Kong/Hospital Authority Hong Kong West Cluster (HKU/HA HKW IRB). Total of 24 patients who underwent curative partial hepatectomy or liver transplantation for HCC between September 2011 and December 2012 at Queen Mary Hospital, Hong Kong, were recruited after written informed consent was obtained. Tumors and adjacent non-tumor liver tissues were collected from the resected specimens. The present data on the characterization of HCC40-PDX and HCC40-CL were new data, while part of the in vitro and in vivo data using freshly isolated GEP-expressing cells had been reported in another study (Addiitonal file 1: Figure S1) [26].

The HCC40-PDX and HCC40-CL original tumor specimen was collected from a 66-year-old Chinese male patient who underwent curative partial hepatectomy. The tumor was 16.0 cm in diameter with venous infiltration, stage II according to the pathological tumor-node-metastasis (pTNM) staging system 2009 version and graded as moderately differentiated. The patient was seronegative for hepatitis B virus (HBV: HBsAg and HBsAb) and hepatitis C virus (HCV: HCVab), and serum α-fetoprotein (AFP) 30286 ng/mL. Intrahepatic and extrahepatic recurrence were observed, and the overall and disease-free survival time were 2.3 and 1.3 months, respectively.

### PDX and cell line establishment

Tumor specimen dissociation, spheroid formation and the subsequent differentiation into adherent cells, and the in vivo tumorigenicity of patients’ tumors were described previously [24, 26]. Briefly, tumor tissues were digested into disaggregated cells by collagenase and then sorted based on their surface GEP by magnetic cell sorting (Miltenyi Biotec, Bergisch Gladbach, Germany) as previously described [23, 26], and the GEP-enriched cells were subject to in vivo PDX and in vitro cell line establishment.

For in vivo PDX establishment, GEP-enriched cells were inoculated subcutaneously into immunocompromised NOD/SCID mice with matrigel (50 %, v/v) (BD Biosciences, San Jose, CA). Xenograft tumors were harvested and passaged when their diameters reached 10 mm. For PDX derived from patient #40, serial xenografts could be generated for more than 10 passages and this line of PDX was designated as HCC40-PDX. HCC40-PDX were cryopreserved at different passages in freezing medium containing 50 % AMEM, 40 % FBS and 10 % DMSO, and stored in liquid nitrogen. After thawing, HCC40-PDX could be propagated in mice without noticeable change in growth rate.

For in vitro cell line establishment, cells were seeded either onto gelatin-coated plate with hepatocyte culture medium (HCM) (Lonza, Basel, Switzerland) according to our previously optimized protocol [24], or ultra-low attachment plate with previously described serum-free and stem cell-promoting medium for spheroid formation [26] to increase the success rate. When spheroids formed, they were dissociated into disaggregated cells and seeded onto culture plate in AMEM supplemented with 10 % FBS. Cells attached and grew into adherent monolayer. The cells derived from patient #40 propagated and were passaged for more than 50 generations hereafter, and this cell line was designated as HCC40-CL. A split ratio of 1:1–1:3 was applied in the early passages (passage 1–5), thereafter increased to 1:10. Cells were collected at different passages and put in freezing medium and stored in liquid nitrogen. After thawing, the cells could be propagated in culture without noticeable change in morphology and growth rate.

### Immunohistochemical (IHC) staining

IHC staining was performed using the Dako Envision Plus System (Dako, Glostrup, Denmark) as previously described [27]. Tissue sections were stained with the mouse anti-human p53, rabbit anti-human HBV core antigen, mouse anti-human HBV surface antigen, AFP (Dako), Ki-67 (BD Biosciences), and equal amount of mouse or rabbit isotype controls (Sigma-Aldrich, St. Louis, MO).

### Immunofluorescence staining and flow cytometric analysis

Cells were permeabilized with ice-cold 0.1 % saponin and then incubated with mouse anti-human albumin, AFP (R&D systems, Minneapolis, MN), or equal amount of mouse IgG isotype (Sigma-Aldrich). Cells were washed with 0.1 % saponin and then stained with PE-conjugated anti-mouse IgG secondary antibody (Dako). After washings, cells were subject to flow cytometric analysis. Results were expressed as percentage of positive cells, after subtracting the non-specific background signal (isotype control).

### Morphological examination and growth kinetics

Cells were routinely monitored using phase-contrast microscope and photographed. Cells from passage 20 were studied to measure the population doubling time, which was assessed by 3-(4,5-dimethylthiazol-2-yl)-2,5-diphenyltetrazolium bromide (MTT) assay for 5 consecutive days.

### Wound healing assay

Cells were seeded onto a 6-well culture plate and incubated for 24 h. A wound was then made by scraping the cell monolayer with a 20 μL pipette tip. Cells were rinsed with PBS and cultured for 3 days. Cell movement toward the wound was observed under a phase-contrast microscope and photographed every 24 h.

### Short tandem repeat (STR) analysis

STR analysis was performed as previously described [24]. DNA samples of the HCC40-CL at passage 20, HCC40-PDX at passage 10, primary tumor and the adjacent non-tumor liver tissue from patient #40 were subjected to DNA fingerprinting analysis using the AmpF/STR Identifiler Plus PCR Amplification Kit (ThermoFisher Scientific Ltd., Waltham, MA).

### Western blot analysis

Total protein was extracted with cell lysis buffer (Cell Signaling Technology, Boston, MA) in the presence of complete protease inhibitor cocktail (Roche, Mannheim, Germany) and separated in 8–10 % SDS PAGE gel. Proteins were then electro-transferred onto polyvinylidene difluoride membranes, subsequently incubated with the mouse anti-human p53 (Dako), E-cadherin (BD Biosciences), β-actin (Sigma-Aldrich), detected by horseradish peroxidase-labeled secondary antibodies, and visualized with Enhanced Chemiluminescence Western Blotting Detection Kit (Amersham Biosciences, Piscataway, NJ).

### *TP53* mutational analysis

DNA samples of the original tumor and the adjacent non-tumor liver tissue from the patient #40, HCC40-CL cells and HCC40-PDX were subjected to direct DNA sequencing for exons 4-9 in p53 as previously described [24].

### In vivo tumorigenicity in immunodeficient mice

The study protocol was approved by and performed in accordance with the Committee of the Use of Live Animals in Teaching and Research at the University of Hong Kong. HCC40-CL cells (passage 20) were harvested, washed, and resuspended in plain AMEM medium. 1 × 10^6^ cells were inoculated subcutaneously into the right flank of each NOD/SCID mouse (4 weeks old). The mice were examined every week for the development of tumors and tumor-bearing mice were sacrificed when tumors were approximately 1 cm in diameters.

### Statistical analyzes

All data were expressed as mean values ± standard deviation (SD) from at least three independent experiments. Differences between groups were assessed by the Student’s t test. A probability (p) <0.05 was considered significantly different. All analyzes were performed using the statistical software GraphPad Prism for Windows, Version 6.00 (GraphPad Software, CA).

## Results

### PDX and primary cell line establishment from fresh HCC tumor tissues

Fresh tumor tissues from 24 HCC patients were included for PDX and cell line establishment. We previously demonstrated that GEP expression was positively correlated with the viability of freshly isolated hepatocytes and the success rate of primary culture establishment [24]. Therefore, GEP-expressing cells were sorted in order to increase the success rate of PDX and cell line establishment. GEP-enriched cells were then subject to both in vivo and in vitro establishment protocols. The in vitro spheroid formation and differentiation ability, and the in vivo tumorigenicity of GEP-expressing cells were described [26], and the workflow of PDX and cell line establishment was illustrated in Additional file 1: Figure S1.

PDXs and cell lines were generated from the tumor specimens of 4 and 3 HCC cases, respectively. Among these successful cases, cells from HCC40 could generate both PDX and primary cell line successfully. Both models were able to propagate for over 10 generations and were then chosen for further characterization. The PDX and its matching cell line were designated as HCC40-PDX and HCC40-CL, respectively (Additional file 1: Figure S1).

### Immunohistochemical characterization of HCC40-PDX

**Fig. 1:**
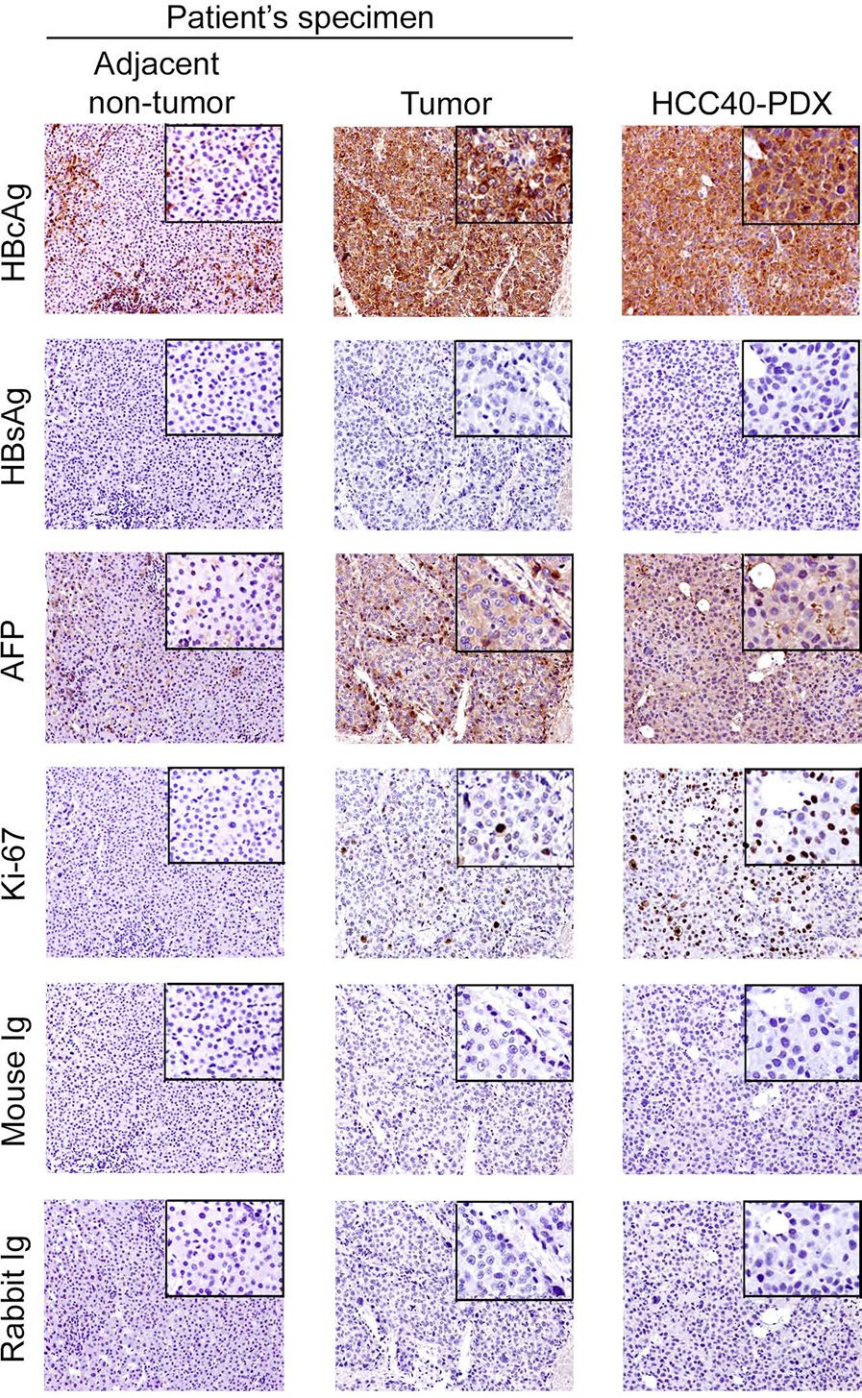
IHC characterization of HCC40-PDX. Paraffin-embedded tissue sections of adjacent non-tumor liver tissue and tumor specimen and HCC40-PDX were stained for HBV core antigen (HBcAg), HBV surface antigen (HBsAg), AFP, Ki-67, and the corresponding IgG controls. The tissue sections were then counterstained with haematoxylin. Magnification: 200×, 400×

IHC staining was performed to compare HCC40-PDX with the original tumor and adjacent non-tumor liver tissue of patient #40. HBV surface antigen (HBsAg) was not detectable in the tumor and non-tumor liver tissue, nor in HCC40-PDX, corroborating that the patient is HBsAg seronegative. Nonetheless, strong HBV core antigen (HBcAg) was detectable in the majority of HCC cells in HCC40-PDX and the primary HCC specimen, and less intense in the adjacent non-tumor liver tissue. Active proliferation indicated by Ki-67 stain and moderate AFP expression was demonstrated in both the primary HCC specimen and HCC40-PDX (Fig. 1).

### Phenotypic and functional characterization of HCC40-CL cells

**Fig. 2:**
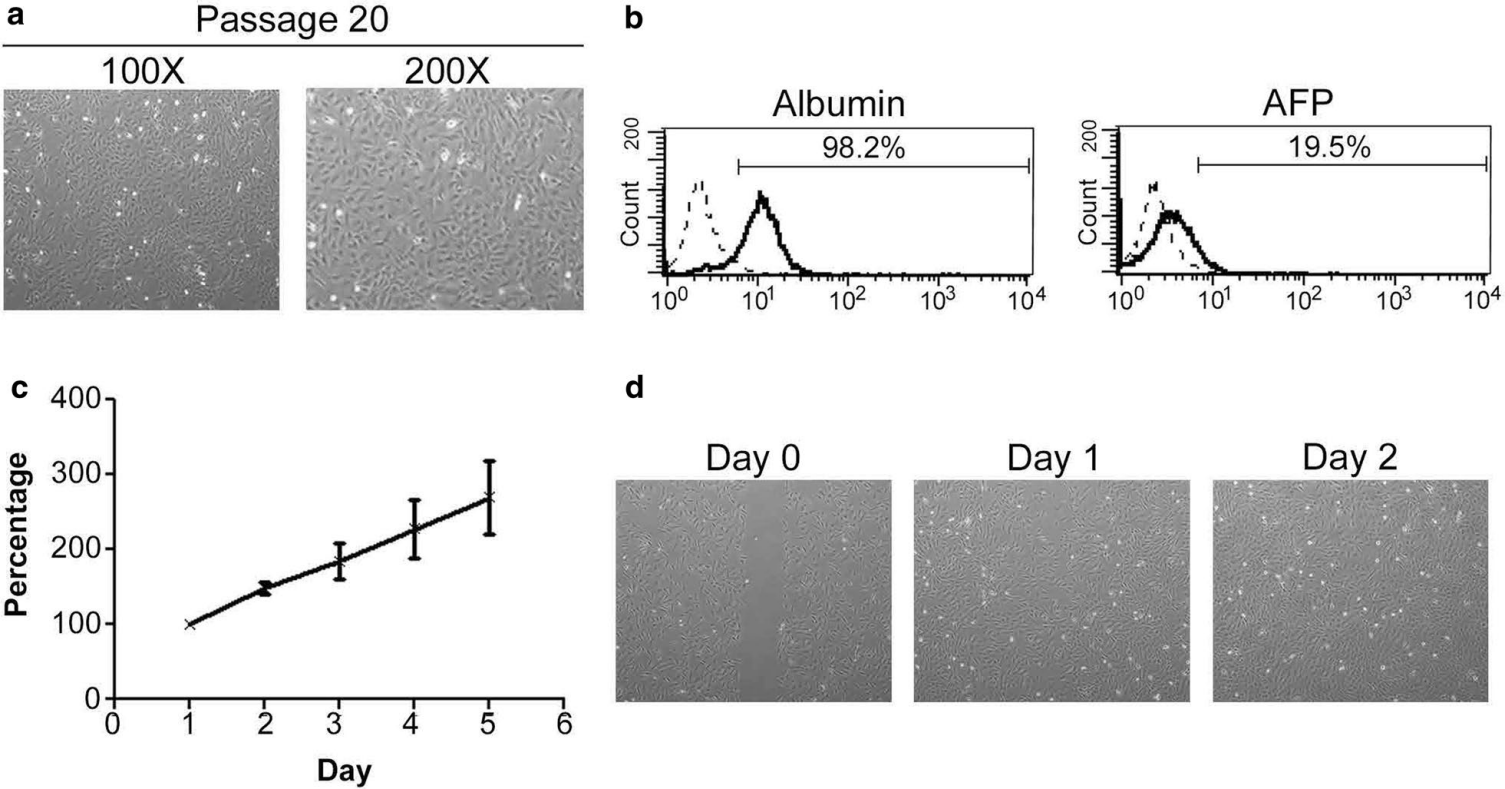
Phenotypic and functional characterization of HCC40-CL. **a** Phase-contrast microscopy images of HCC40-CL cells at passages 20 (Magnification: 100×, 200×). **b** Albumin and AFP levels of HCC40-CL cells were measured by flow cytometry. Percentages of albumin+ and AFP+ cells were indicated in the *histograms*. *Dotted line*: isotype control; *Solid line*: Albumin or AFP antibody. **c** Growth curve of HCC40-CL cells (passage 20) as determined by MTT. **d** Wound healing assay showing the migration ability of HCC40-CL cells. A wound was made by scraping the cell monolayer with a 20 μL pipette tip. Cell movement was photographed and observed under phase-contrast microscopy

HCC40-CL cells were passaged for more than 50 generations since establishment. The cells grew as adherent monolayer with epithelial morphology and maintained consistent morphology along passages (Fig. 2a; Additional file 2: Figure S2). Flow cytometric analysis showed that 98.2 % of the cells were albumin-positive, confirming that HCC40-CL cells were in the hepatic lineage. Besides, AFP positive cells by flow cytometry (Fig. 2b) corroborated the AFP staining pattern by immunohistochemistry (Fig. 1) and high serum AFP levels. Growth curve of HCC40-CL cells (passage 20) was shown in Fig. 2c. The population doubling time of HCC40-CL was approximately 43 h. Wound healing assay was performed to assess the migration ability of HCC40-CL cells. The cells started to migrate 1 day after the wound was made, and the wound healing was completed after 2 days (Fig. 2d). The migration ability of HCC40-CL cells echoed the histological observation that venous infiltration was present in the primary specimen.

### Tumorigenicity and metastatic potential of HCC40-CL cells in immunodeficient mice

**Fig. 3:**
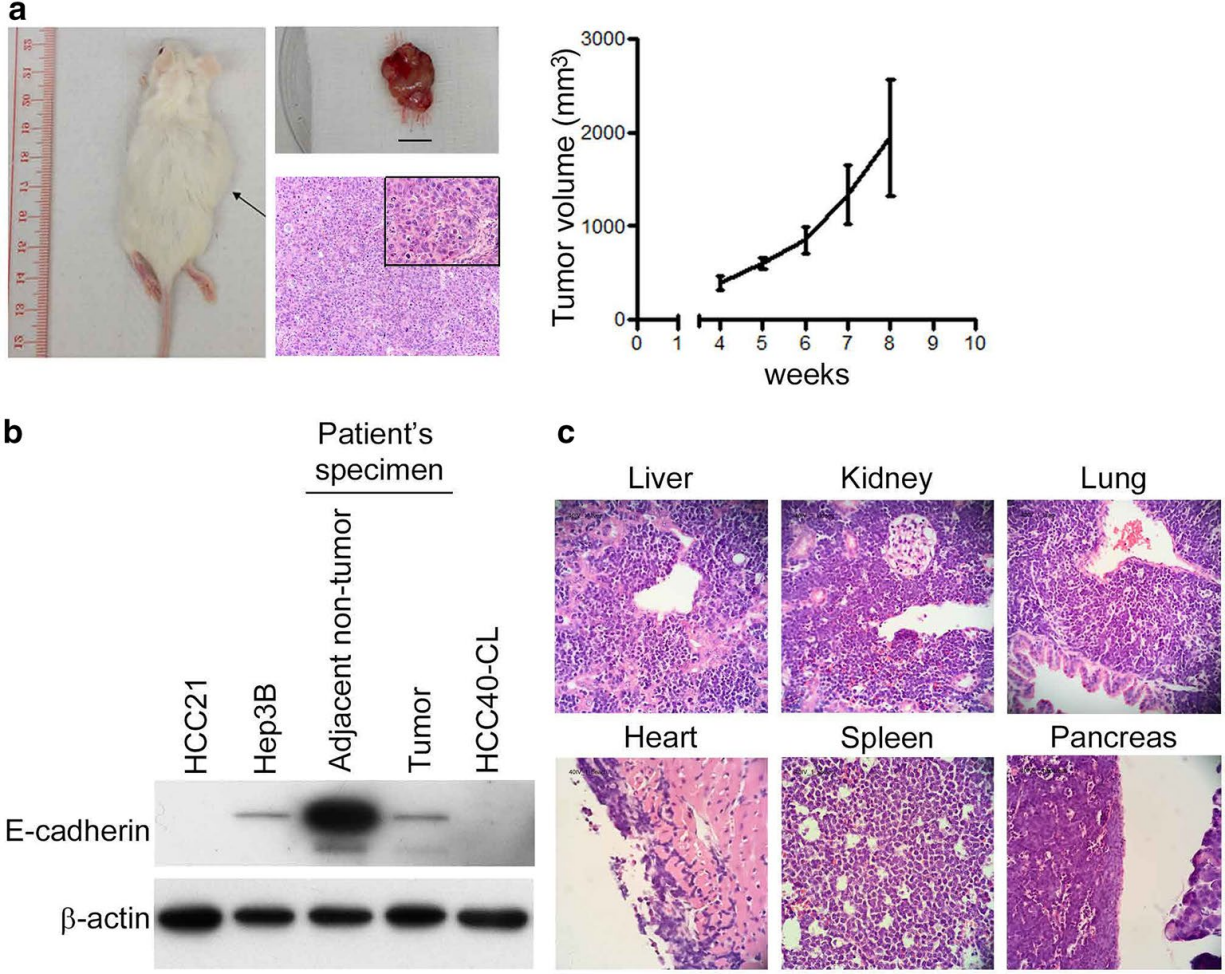
Tumorigenicity and metastatic potential of HCC40-CL cells in NOD/SCID mice. **a** NOD/SCID mice inoculated subcutaneously with 1 × 10^6^ HCC40-CL cells at 8 weeks post-injection. The *middle panel* showed the corresponding xenograft tumor derived from HCC40-CL cells (*scale bar* 10 mm) and H&E staining of the paraffin-embedded tissue section (Magnification: 200×, 400×). The *right panel* showed the growth curve of HCC40-CL cell-derived xenografts (n = 4). **b** Protein expression of E-cadherin in HCC21 (negative control), Hep3B (positive control), adjacent non-tumor liver tissue and tumor specimen from patient HCC40, and HCC40-CL cells. β-actin served as loading control. **c** HCC40-CL cells were injected intravenously into NOD/SCID mice. H&E staining showed extensive metastases observed in the liver, kidney, lung, heart, spleen and pancreas of the mice

HCC40-CL cells (1 × 10^6^) were inoculated subcutaneously into 4 NOD/SCID mice to assess its in vivo tumorigenicity. About 4 weeks after injection, visible tumors developed in all mice at the site of inoculation (Fig. 3a), indicating that HCC40-CL cells were tumorigenic. Tumors were dissected, and H&E staining was performed on the paraffin-embedded tissue section (Fig. 3a). The growth curve of tumors was shown in Fig. 3a (right panel). Besides, IHC analysis demonstrated the expression of HBcAg, AFP and Ki-67, but not HBsAg, in the HCC40-CL xenograft (Additional file 3: Figure S3). Such expression pattern was consistent with that observed in original tumor (Fig. 1).

To assess the metastatic potential, we measured the expression of E-cadherin, an adhesion molecule essential for stable cell connection and was known to be down-regulated for metastasis [28–30]. Western blot analysis showed that E-cadherin protein, which was present in the patient’s non-tumor liver tissue, was down-regulated in the primary tumor specimen and HCC40-CL cells (Fig. 3b). HCC21 is a metastatic primary cell line established by our group previously [24], and was included as a negative control for E-cadherin protein expression; while Hep3B, a non-metastatic HCC cell line, served as positive control. We further investigated the metastatic potential of HCC40-CL cells by injecting the cells (1 × 10^6^) intravenously into NOD/SCID mice. Extensive metastases were observed in liver, kidney, lung, heart, spleen and pancreas (Fig. 3c).

### STR profile analysis of HCC40-PDX and HCC40-CL cells

The DNA samples extracted from the primary tumor specimen and adjacent non-tumor liver tissue, HCC40-PDX and HCC40-CL were subject to STR analysis. A total of 15 STR loci (CSF1P0, D2S1338, D3S1358, D5S818, D7S820, D8S1179, D13S317, D16S539, D18S51, D19S433, D21S11, FGA, TH01, TPOX, vWA) were co-amplified in each sample. *AMLEO* locus at the sex chromosomes was also examined. The data were analyzed and allele(s) of each locus were determined (Table 1). The STR profiles demonstrated that HCC40-PDX and HCC40-CL were derived from the primary specimen HCC40. Notably, additional genetic aberrations were observed at loci CSF1P0 and D16S539 in both HCC40-PDX and HCC40-CL along the establishment.

**Table 1:** STR profiles of adjacent non-tumor liver tissue and tumor specimen, HCC40-PDX and HCC40-CL

Locus	Adjacent non-tumor	Tumor	HCC40-PDX	HCC40-CL
D8S1179	13	13	13	13
D21S11	28, 32.2	28, 32.2	28, 32.2	28, 32.2
D7S820	10, 11	10, 11	10, 11	10, 11
CSF1PO	10, 11	10, 11	*10, 11, 12*	*10, 11, 12*
D3S1358	15, 16	15, 16	15, 16	15, 16
TH01	6, 9	6, 9	6, 9	6, 9
D13S317	8, 9	8, 9	8, 9	8, 9
D16S539	10, 12	10, 12	*12*	*12*
D2S1338	18, 21	18, 21	18, 21	18, 21
D19S433	13, 15	13, 15	13, 15	13, 15
vWA	14, 19	14, 19	14, 19	14, 19
TPOX	8, 9	8, 9	8, 9	8, 9
D18S51	17, 18	17, 18	17, 18	17, 18
AMELOGENIN	X, Y	X, Y	X, Y	X, Y
D5S818	11, 13	11, 13	11, 13	11, 13
FGA	22, 25	22, 25	22, 25	22, 25

Differences were italicized

### TP53 mutational analysis of HCC40-PDX and HCC40-CL cells

**Fig. 4:**
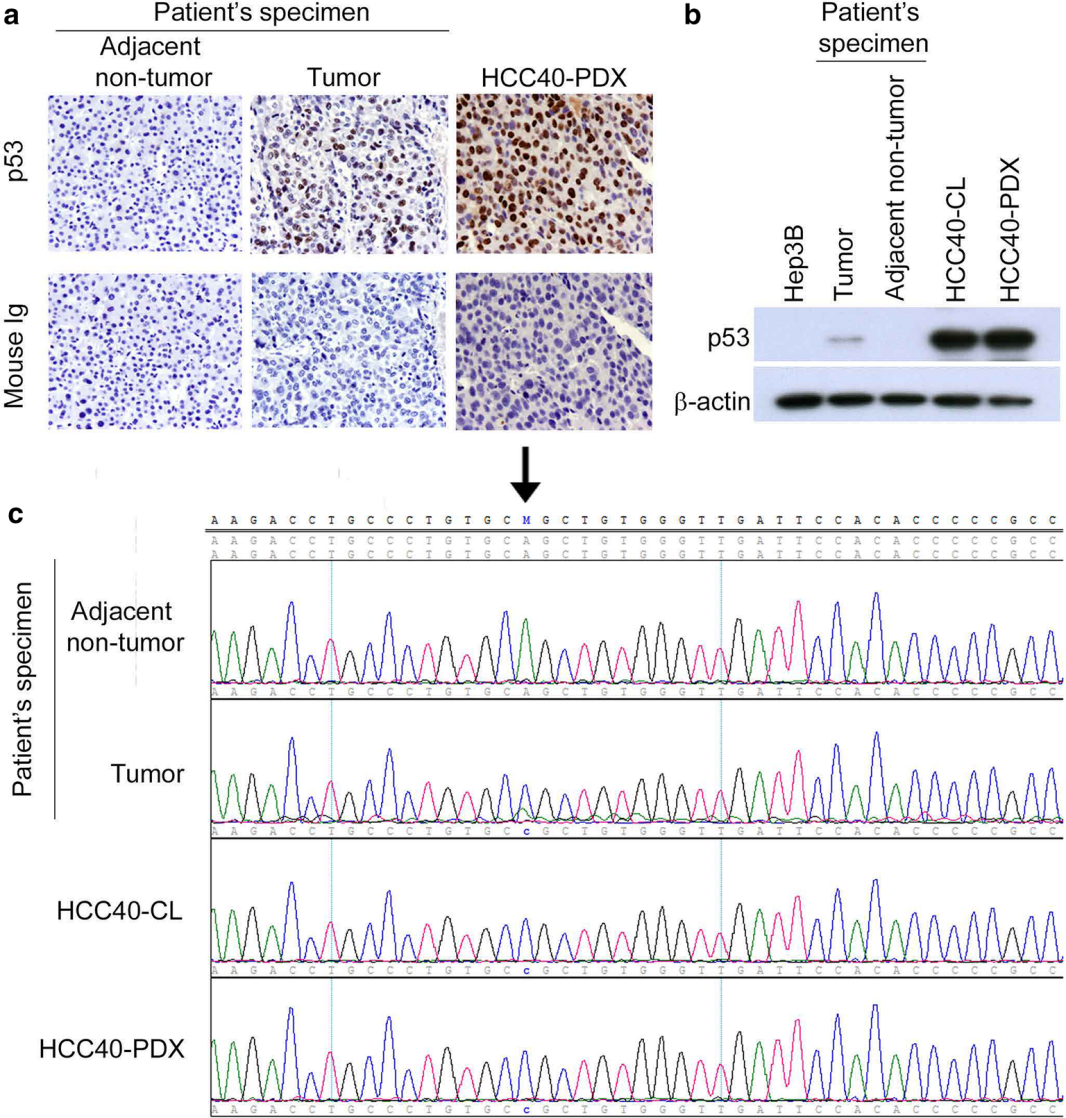
TP53 mutational analysis in original tumor and adjacent non-tumor liver tissue, HCC40-CL and HCC40-PDX. **a** IHC staining of p53 in adjacent non-tumor liver tissue and tumor specimen, and HCC40-PDX. Magnification: 400×. **b** Expression of p53 protein in Hep3B (p53 null), primary tumor specimen, adjacent non-tumor liver tissue, HCC40-CL and HCC40-PDX by western blot. β-actin served as loading control. **c** Sequencing analysis of TP53 gene revealed a point mutation at codon 104 of exon 5 (CAG → CCG) (Gln → Pro) in tumor specimen, HCC40-CL, and HCC40-PDX, but not in adjacent non-tumor liver tissue

IHC staining showed that p53 nuclear accumulation in the primary tumor and HCC40-PDX, but not in adjacent non-tumor liver tissue (Fig. 4a). Western blot analysis demonstrated the presence of p53 protein in the primary tumor, HCC40-CL and HCC40-PDX, but not in non-tumor adjacent liver tissue (Fig. 4b). Subsequent analysis by DNA sequencing further consolidated a sporadic point mutation at codon 104 of exon 5 (CAG → CCG) (Gln → Pro) in the primary tumor specimen, and identical mutation was observed in both HCC40-CL and HCC40-PDX (Fig. 4c).

## Discussion

PDX models are known to preserve most of the key biological properties of their primary tumors and remain stable across passages. These models are highly predictive of clinical outcomes and therefore offer a potential for personalizing cancer treatments as well as assisting clinical trial designs [10–15]. The responses to targeted therapeutic agents have been reported to be highly correlated between PDX models and patients [10–16]. These models would be useful for testing the FDA-approved targeted drug sorafenib [2] and new target at preclinical phase including GEP antibodies for growth inhibition and chemo-sensitization [31, 32]. In this study, we established a PDX model from a patient with early staged and moderately differentiated HCC. Our PDX model, HCC40-PDX, showed remarkable congruence in the biological phenotypes and molecular details of the primary tumor. The models were authenticated by STR analysis, and both could be cryopreserved, so that stable supply of the models for drug and other assays could be ensured. It was demonstrated that serial propagation in mice did not significantly change the biological characteristics of xenograft tumors. Studies that compared the response to drug treatments of PDXs from different passages demonstrated stable response rates across generations, further supporting the phenotypic stability of these models [11, 33] and making them a useful tool for studying pathogenesis of HCC and its therapeutic strategy.

In addition to PDX models, their matching cell lines are also valuable tools as they allow high throughput drug screening and genetic manipulation for in depth mechanistic study. Here, we established and characterized a matching cell line from the same patient from which HCC40-PDX was derived. The matching cell line, HCC40-CL, was authenticated, characterized and showed congruence in the p53 mutational status with the primary tumor. Importantly, this cell line possessed metastatic ability. HCC has been reported with high incidence of metastasis, which is a major obstacle to HCC treatment [34]. The underlying mechanism of metastasis in HCC is not well-characterized, which is probably due to the lack of appropriate models for the related studies. Here, we showed that intravenous injection of HCC40-CL led to extensive metastases in immunocompromised mice, indicating the metastatic ability of the cells. In current study, we assessed the expression of E-cadherin in the original tumor and liver specimens, and HCC40-CL cells. E-cadherin is a cell adhesion molecule essential for establishing stable intercellular adherent junctions, and its down-regulation is associated with infiltrative growth and metastasis in various cancers including HCC [35, 36]. We showed that E-cadherin in original tumor specimen was reduced when compared to the adjacent non-tumor liver tissue, and the down-regulation was retained in HCC40-CL cells, implying a metastatic potential of in both original tumor specimen and HCC40-CL cells. For HCC40-PDX subcutaneous inoculation, however, no metastasis was observed in the recipient mice. Independent research groups have reported that subcutaneously transplanted tumors were less prone to metastasize either regionally or distally [37, 38]. Orthotopic implantations were shown to form vascularized xenografts more readily and therefore higher frequency of spontaneous distant metastasis could be observed [38]. Besides, the organ site corresponding to the tumor origin would allow the tumor to behave more similarly to the original tumor. Therefore, further investigation on the metastatic potential of HCC40-PDX should be performed using orthotopic model.

p53 is frequently mutated and overexpressed in HCC [39]. p53 alterations are reported to correlate with the aggressiveness of HCC, including tumor differentiation, vascular invasion and tumor stage [40, 41]. Missense mutations leading to amino acid substitutions are common mutation in TP53 [42]. Here, we showed a point mutation in the TP53 gene at codon 104 of exon 5 (CAG → CCG) (Gln → Pro) in tumor specimen, and the mutation was retained in both HCC40-CL cells and HCC40-PDX. This mutation has not been reported in HCC previously. However, this mutation might result in the presence of aberrant protein with increased stability and nuclear accumulation in the cells, similarly as other p53 mutations [42]. This is supported by the strong nuclear protein expression of p53 in HCC40-PDX and the primary tumor specimen, when compared to the adjacent non-tumor liver tissue (Fig. 4a).

In current study, disaggregated tumor cells from HCC patients were sorted for hepatic cancer stem cell marker GEP to increase the cell viability and facilitate the PDX and cell line establishment. We previously showed that GEP-expressing cells possess CSC properties in HCC [23]. Asymmetric cell division is a defining CSC property, which enables them to simultaneously perpetuate themselves i.e. self-renew, and generate differentiated progenies [43]. Indeed, we showed in a separate study that transplantation of sorted GEP^high^ cells (GEP + cells: >80 %) into immunocompromised mice could generate heterogeneous tumor mass consisting of both GEP+ and GEP− cells, in which GEP levels were found to return to the level of the original tumors from which they were derived [26]. Similar phenomenon has also been observed in other HCC CSC markers such as CD24 and CD133 [44, 45]. Therefore, although GEP-expressing cells were enriched for PDX and cell line establishment to increase success rate, their levels would return to recapitulate those of the original tumors, and would not cause bias to the cellular composition due to the CSC nature of GEP-expressing cells.

## Conclusions

We have established a PDX model and the matching primary cell line from an early-staged and moderately differentiated HCC. Our newly established models will not only aid in the development of new therapeutic strategies, but also in gaining insight in the mechanisms underlying how the tumors respond to therapeutic agents. This, in turn, can shed light on the molecular pathogenesis of HCC. Future work includes expanding the pool of PDXs, together with their matching cell lines, to examine the heterogeneous HCC.

## Additional files

10.1186/s12935-016-0322-5 PDX and cell line establishment from fresh HCC tumor specimens. Experimental workflow of establishing PDXs and primary cell lines from HCC patients was illustrated. Fresh tumor tissues were collected from 24 HCC patients and subject to enzymatic digestion by collagenase to release disaggregated cells. Only cells with viability >70% (11 out of 24 cases) were subject to subsequent cell sorting for GEP-expressing cells. For *in vivo* PDX establishment, GEP-enriched cells were inoculated with 50% matrigel (v/v) subcutaneously at the dorsal region of the trunk of NOD/SCID mice (11 cases). 4 out of 11 cases generated xenograft tumors. NOD/SCID mice inoculated with cells from HCC40 (10 weeks post-inoculation) was shown, while the lower panel shows the corresponding xenograft tumor (HCC40-PDX). For *in vitro* primary cell line establishment, cells were either seeded onto gelatin-coated plate in hepatocyte culture medium (HCM) (11 cases), or ultra-low attachment plate in serum-free, stem cell-promoting medium for spheroid formation (3 cases). 3 out of 11 cases generated cells that could attach and grow within 1 month. The phase contrast microscopy image showed the cells derived from patient HCC40. For spheroid culture, spheroids formed in all 3 cases after 1-month culture. Spheroids derived from patient HCC40 were shown. The spheroids were dissociated to disaggregated cells, which were then seeded onto culture plate in serum-supplemented medium to induce differentiation of cells to grow into adherent monolayer. The adherent cells grew from spheroids derived from patient #40 was designated as HCC40-CL.
10.1186/s12935-016-0322-5 Morphology of HCC40-CL cells in early and late passages. Phase contrast microscopy images showing the morphology of HCC40-CL cells at passages 5, 15 and 40. Magnification: 100×.
10.1186/s12935-016-0322-5 Phenotypic characterization of HCC40-CL xenograft tumor. Paraffin-embedded tissue sections of HCC40-CL xenograft were stained for HBcAg, HBsAg, AFP, Ki-67 and corresponding isotype controls. The tissue sections were then counterstained with hematoxylin. Magnification: 200×, 400×.

## Abbreviations

AFP: alpha-fetoprotein
GEP: granulin-epithelin precursor
HCC: hepatocellular carcinoma
PDX: patient-derived xenograft
STR: short tandem repeat
CSC: cancer stem cell
