# Supplementary material for: Comprehensive characterization of the patient-derived xenograft and the paralleled primary hepatocellular carcinoma cell line
Source: Cancer Cell Int. 2016 Jun 8;16:41. doi: 10.1186/s12935-016-0322-5 (PMC4898407; doi:10.1186/s12935-016-0322-5)
Supplement: Supplementary file 1 — 10.1186/s12935-016-0322-5 PDX and cell line establishment from fresh HCC tumor specimens. Experimental workflow of establishing PDXs and primary cell lines from HCC patients was illustrated. Fresh tumor tissues were collected from 24 HCC patients and subject to enzymatic digestion by collagenase to release disaggregated cells. Only cells with viability >70% (11 out of 24 cases) were subject to subsequent cell sorting for GEP-expressing cells. For in vivo PDX establishment, GEP-enriched cells were inoculated with 50% matrigel (v/v) subcutaneously at the dorsal region of the trunk of NOD/SCID mice (11 cases). 4 out of 11 cases generated xenograft tumors. NOD/SCID mice inoculated with cells from HCC40 (10 weeks post-inoculation) was shown, while the lower panel shows the corresponding xenograft tumor (HCC40-PDX). For in vitro primary cell line establishment, cells were either seeded onto gelatin-coated plate in hepatocyte culture medium (HCM) (11 cases), or ultra-low attachment plate in serum-free, stem cell-promoting medium for spheroid formation (3 cases). 3 out of 11 cases generated cells that could attach and grow within 1 month. The phase contrast microscopy image showed the cells derived from patient HCC40. For spheroid culture, spheroids formed in all 3 cases after 1-month culture. Spheroids derived from patient HCC40 were shown. The spheroids were dissociated to disaggregated cells, which were then seeded onto culture plate in serum-supplemented medium to induce differentiation of cells to grow into adherent monolayer. The adherent cells grew from spheroids derived from patient #40 was designated as HCC40-CL. [file 12935_2016_322_MOESM1_ESM.docx]

**Figure S1. PDX and cell line establishment from fresh HCC tumor specimens.** Experimental workflow of establishing PDXs and primary cell lines from HCC patients was illustrated. Fresh tumor tissues were collected from 24 HCC patients and subject to enzymatic digestion by collagenase to release disaggregated cells. Only cells with viability >70% (11 out of 24 cases) were subject to subsequent cell sorting for GEP-expressing cells. For *in vivo* PDX establishment, GEP-enriched cells were inoculated with 50% matrigel (v/v) subcutaneously at the dorsal region of the trunk of NOD/SCID mice (11 cases). 4 out of 11 cases generated xenograft tumors. NOD/SCID mice inoculated with cells from HCC40 (10 weeks post-inoculation) was shown, while the lower panel shows the corresponding xenograft tumor (HCC40-PDX). For *in vitro* primary cell line establishment, cells were either seeded onto gelatin-coated plate in hepatocyte culture medium (HCM) (11 cases), or ultra-low attachment plate in serum-free, stem cell-promoting medium for spheroid formation (3 cases). 3 out of 11 cases generated cells that could attach and grow within 1 month. The phase contrast microscopy image showed the cells derived from patient HCC40. For spheroid culture, spheroids formed in all 3 cases after 1-month culture. Spheroids derived from patient HCC40 were shown. The spheroids were dissociated to disaggregated cells, which were then seeded onto culture plate in serum-supplemented medium to induce differentiation of cells to grow into adherent monolayer. The adherent cells grew from spheroids derived from patient #40 was designated as HCC40-CL.
