# Supplementary material for: Comprehensive characterization of the patient-derived xenograft and the paralleled primary hepatocellular carcinoma cell line
Source: Cancer Cell Int. 2016 Jun 8;16:41. doi: 10.1186/s12935-016-0322-5 (PMC4898407; doi:10.1186/s12935-016-0322-5)
Supplement: Supplementary file 2 — 10.1186/s12935-016-0322-5 Morphology of HCC40-CL cells in early and late passages. Phase contrast microscopy images showing the morphology of HCC40-CL cells at passages 5, 15 and 40. Magnification: 100×. [file 12935_2016_322_MOESM2_ESM.docx]

**Figure S2. Morphology of HCC40-CL cells in early and late passages.** Phase contrast microscopy images showing the morphology of HCC40-CL cells at passages 5, 15 and 40. Magnification: 100X.
