# Supplementary material for: Comprehensive characterization of the patient-derived xenograft and the paralleled primary hepatocellular carcinoma cell line
Source: Cancer Cell Int. 2016 Jun 8;16:41. doi: 10.1186/s12935-016-0322-5 (PMC4898407; doi:10.1186/s12935-016-0322-5)
Supplement: Supplementary file 3 — 10.1186/s12935-016-0322-5 Phenotypic characterization of HCC40-CL xenograft tumor. Paraffin-embedded tissue sections of HCC40-CL xenograft were stained for HBcAg, HBsAg, AFP, Ki-67 and corresponding isotype controls. The tissue sections were then counterstained with hematoxylin. Magnification: 200×, 400×. [file 12935_2016_322_MOESM3_ESM.docx]

**Figure S3. Phenotypic characterization of HCC40-CL xenograft tumor.**

Paraffin-embedded tissue sections of HCC40-CL xenograft were stained for HBcAg, HBsAg, AFP, Ki-67 and corresponding isotype controls. The tissue sections were then counterstained with hematoxylin. Magnification: 200X, 400X.
